# Emerging Functions of Human IFIT Proteins in Cancer

**DOI:** 10.3389/fmolb.2019.00148

**Published:** 2019-12-19

**Authors:** Vijaya Kumar Pidugu, Hima Bindu Pidugu, Meei-Maan Wu, Chung-Ji Liu, Te-Chang Lee

**Affiliations:** ^1^Institute of Biomedical Sciences, Academia Sinica, Taipei, Taiwan; ^2^Department of Public Health, School of Medicine, College of Medicine, Taipei Medical University, Taipei, Taiwan; ^3^Department of Oral and Maxillofacial Surgery, Mackay Memorial Hospital, Taipei, Taiwan; ^4^Institute of Pharmacology, National Yang-Ming University, Taipei, Taiwan

**Keywords:** IFIT, cancer, TPRs, OSCC, progression, metastasis, drug resistance

## Abstract

Interferon-induced protein with tetratricopeptide repeats (IFIT) genes are prominent interferon-stimulated genes (ISGs). The human IFIT gene family consists of four genes named *IFIT1, IFIT2, IFIT3*, and *IFIT5*. The expression of IFIT genes is very low in most cell types, whereas their expression is greatly enhanced by interferon treatment, viral infection, and pathogen-associated molecular patterns (PAMPs). The proteins encoded by IFIT genes have multiple tetratricopeptide repeat (TPR) motifs. IFIT proteins do not have any known enzymatic roles. However, they execute a variety of cellular functions by mediating protein-protein interactions and forming multiprotein complexes with cellular and viral proteins through their multiple TPR motifs. The versatile tertiary structure of TPR motifs in IFIT proteins enables them to be involved in distinct biological functions, including host innate immunity, antiviral immune response, virus-induced translation initiation, replication, double-stranded RNA signaling, and PAMP recognition. The current understanding of the IFIT proteins and their role in cellular signaling mechanisms is limited to the antiviral immune response and innate immunity. However, recent studies on IFIT protein functions and their involvement in various molecular signaling mechanisms have implicated them in cancer progression and metastasis. In this article, we focused on critical molecular, biological and oncogenic functions of human IFIT proteins by reviewing their prognostic significance in health and cancer. Research suggests that IFIT proteins could be novel therapeutic targets for cancer therapy.

## Introduction

The interferon-induced protein with tetratricopeptide repeats (IFIT) gene family is well-studied interferon-stimulating genes (ISGs) for their antiviral properties (Fensterl and Sen, 2015). IFIT gene products are cytoplasmic proteins with no known enzymatic activity, but all of them share special structural motifs known as tetratricopeptide repeats (TPRs). The TPR motifs consist of helix-turn-helix structures that can make multiple protein complexes in the cells (Allan and Ratajczak, 2011). IFIT proteins are involved in a variety of biological processes, such as cell proliferation, migration, virus-induced translation initiation, replication and double-stranded RNA signaling (Fensterl and Sen, 2011). Most cell types without stimulation do not express IFIT genes at high levels, whereas the transcription of IFIT genes is rapidly induced by interferon (IFN) treatment and viral infection. The antiviral functions of human IFIT proteins have been extensively demonstrated in many research studies (Fensterl and Sen, 2011, 2015; Diamond and Farzan, 2013). Recent studies have implicated IFIT proteins as prognostic markers to determine the clinical outcome of many cancers, such as glioblastoma, hepatocellular carcinoma, breast cancer and pancreatic cancer (Danish et al., 2013; Zhang et al., 2016; Yang et al., 2017; Zhao et al., 2017). During the past several years, we explored the biological and clinical functions of IFIT proteins in oral squamous cell carcinoma (OSCC) (Blot et al., 1988; Lai et al., 2008, 2013; Pidugu et al., 2019b). In this review, we focus on the molecular and clinical significance of IFIT proteins in cancer and discuss their emerging oncogenic roles, with a special emphasis on the progression of human OSCC.

### IFIT Expression in OSCC

Oral cancer is one of the most familiar malignant cancers worldwide, and it is estimated that 3.6% of cancer deaths were due to oral cancer in 2012 (Shield et al., 2017). It is also one of the fastest-increasing malignancies and the fourth leading cause of death in male cancer patients (Liu et al., 2010). OSCC accounts for more than 90% of oral cancers. Betel quid chewing, tobacco use and alcohol consumption are the major risk factors for OSCC (Su et al., 2007). In addition, human papillomavirus (HPV) infection has been reported as an aetiological agent of OSCC (Gillison et al., 2008; Husain and Neyaz, 2017). However, HPV-16-positive patients had better clinical outcomes than patients with HPV-negative tumors (Schwartz et al., 2001). Because of the synergistic tumor-promoting effect of cigarette smoking and alcohol consumption, people with heavy smoking and alcohol consumption habits are more prone to OSCC than those who only smoke or drink (Blot et al., 1988; Haddad and Shin, 2008; Jha, 2009). A high incidence of OSCC has been reported in Asian countries due to the cultural practice of chewing betel nuts (Su et al., 2007; Liu et al., 2010; Krishna Rao et al., 2013). Several studies have reported the relationship between betel nut chewing and increased mortality rate from OSCC (Jeng et al., 2001; Wen et al., 2010). OSCC is usually diagnosed in advanced stages due to ignorance of the patients or inaccurate diagnosis (Scott et al., 2006). OSCC patients with advanced-stage (III and IV) tumors are usually treated with extensive surgery combined with radiation and chemotherapies (Kirita et al., 2012; Noguti et al., 2012). The overall 5 year survival rate of OSCC patients usually varies from 40 to 50% (Markopoulos, 2012). Disappointingly, the 5 year survival rates are low in OSCC patients, and no significant improvement has been seen (Wang et al., 2013). The development of drug resistance by intrinsic molecular mechanisms causes treatment failure. Therefore, it is of fundamental importance to identify the tumor-intrinsic pathways involved in drug resistance and metastasis for the development of effective therapies for the treatment of OSCC patients.

During the course of our research, we found that IFIT protein levels were altered in OSCC patient tissues. However, how and why their expression was induced in OSCC is still unknown. That warrants further investigation. It has been reported that the increased incidence of OSCC in Asian countries is caused by chewing betel quid's (Krishna Rao et al., 2013). Many research studies have documented the association between OSCC progression and betel nut (BN) or betel quid (BQ) chewing (Jeng et al., 2001). The carcinogenic components of the areca nut cause malignant transformation of cells that leads to increased risk for the development of OSCC. Hence, chewing BNs/BQs is considered an independent risk factor for OSCC (Warnakulasuriya et al., 2002; Sharan et al., 2012). As shown in previous studies, areca nut extract treatment resulted in the differential expression of various cellular genes, including *IFIT2*, in human keratinocytes (Lai and Lee, 2006; Lai et al., 2008). Therefore, we speculated that BQ chewing may influence IFIT expression in tumor tissues of OSCC patients. We preliminary found that high *IFIT1, IFIT3*, and *IFIT5* expression levels are significantly associated with betel quid chewing in patients. However, further prospective cohort studies are required to determine the influence of betel quid chewing on IFIT expression in patients with OSCC. The distinct expression of individual IFIT genes in the same cell or tissue is believed to result in non-redundant functions (Terenzi et al., 2007; Wacher et al., 2007). Hence, we hypothesized that individual IFIT proteins may exhibit unique functions depending on the cell type.

### Gene Structure and Transcriptional Regulation of Human IFITs

**Figure 1:**
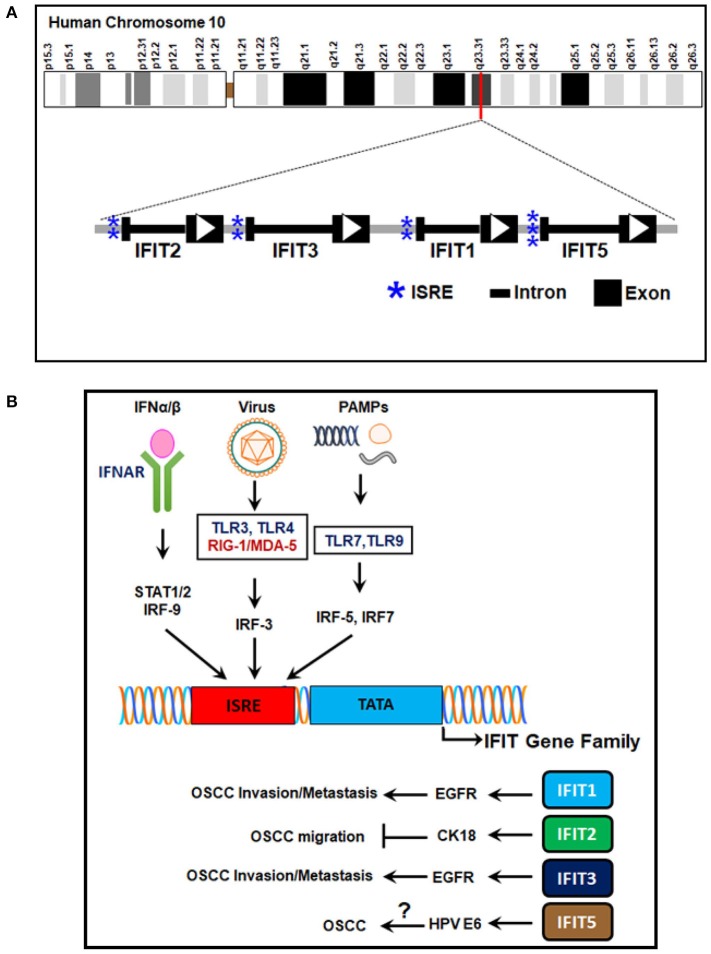
Human IFIT gene family location, structure, and transcriptional regulation. **(A)** The genetic architecture of human IFITs. The human IFIT genes are located on human chromosome 10q23.31. The exons are depicted as black boxes, the introns as dark lines, and ISREs in the promoter regions as blue asterisks. The direction of the open reading frames (ORFs) is designated by white arrowheads. **(B)** Signaling pathways of IFIT gene transcription. The ISREs in the promoter regions of IFIT genes are induced by stimulation of cell surface receptors such as *IFNAR-*α*/*β*, TLR-3, RIG-I/MDA-5, TLR-7*, and *TLR-9* by *IFN-*α*/*β, viral infection, and PAMPs. Transcriptional induction is mediated by various transcription factors, such as *IRF-3, IRF-5, IRF-7*, and *IRF-9*. The encoded proteins (*IFIT1, IFIT2, IFIT3*, and *IFIT5*) regulate OSCC progression. *IFIT1/IFIT3* promote OSCC invasion and metastasis by *EGFR* activation. *IFIT2* inhibits OSCC migration via *CK-18*. IFIT5 expression is correlated with HPV E6 protein in OSCC. However, its mechanism in OSCC is not yet understood. *IFN-*α*/*β, interferon alpha/beta; *IFNAR-*α*/*β, interferon alpha/beta receptors; *STAT1/2*, signal transducer and activator of transcription 1/2; *TLRs*, Toll-like receptors; *RIG-I*, retinoic acid-inducible gene-I; *MDA-5*, melanoma differentiation-associated gene-5; *IRFs*, interferon regulatory factors; *ISRE*, interferon-stimulated responsive elements; *IFIT*, interferon-induced proteins with tetratricopeptide repeats; *EGFR*, epidermal growth factor receptor, *CK-18*, cytokeratin-18; *HPV E6*, human papilloma virus early protein-6; *OSCC*, oral squamous cell carcinoma.

The human IFIT gene family is composed of four genes: *IFIT1, IFIT2, IFIT3*, and *IFIT5*. They are clustered on human chromosome 10q23.31 (Table 1) (Varela et al., 2014). Besides, an untranscribed IFIT1 pseudogene, *IFIT1P* has been identified on human chromosome 13 (Wathelet et al., 1988). The IFIT genes have a simple gene architecture with 2 exons and a promoter. The two exons are separated by an intronic region that extends a few kilobases in length. The first exon is small and is next to interferon-stimulated response elements (ISREs), regulatory elements responsible for IFN treatment. The second exon encodes the protein-coding mRNA sequence (Fensterl and Sen, 2011, 2015; Liu et al., 2013) (Figure 1A). The presence of the ISREs in the promoter regions of IFIT genes could be the reason for their low basal expression and rapid IFN-mediated transcriptional induction (Sarkar and Sen, 2004; Pichlmair et al., 2011). INFs are broadly divided into two groups, type I and type II; type I contains *IFN-α, IFN-*β*, IFN-δ, IFN-ε, IFN-κ, IFN-τ*, and *IFN-ω*, and type II IFNs include *IFN-γ* (Pestka et al., 1987, 2004; Platanias, 2005). *IFN-*α*, -β*, or -*γ* treatment activates the transcription of a multitude of ISGs, including IFIT genes; it was roughly estimated that these number 500–1000 genes, depending upon cell or tissue type (Der et al., 1998; de Veer et al., 2001). It was noted that IFIT proteins are extensively induced by *IFN-*α compared to *IFN-*γ treatment (Der et al., 1998; de Veer et al., 2001). In addition to IFN stimuli, the expression of IFITs can also be triggered by several signaling pathways, such as those of Toll-like receptor 3 (*TLR3*), retinoic acid-inducible gene-I/melanoma differentiation-associated gene-5 (*RIG-I/MDA-5*) and pathogen-associated molecular patterns (PAMPs) (Fensterl and Sen, 2011; Diamond and Farzan, 2013) (Figure 1B).

**Table 1:** Synopsis of all available gene database information and synonyms for Human IFIT genes.

**Gene name**	**Entrez gene ID**	**HGNC ID**	**Ensemble ID**	**OMIM ID**	**Alternate names**
*IFIT1*	3434	5407	ENSG00000185745	147690	*IFI-56K, IFNAI1, IFIT-1, G10P1, IFI56, ISG56, P56, IFI56, RNM561, C56, Hup56*
*IFIT2*	3433	5409	ENSG00000119922	147040	*ISG54K, IFI-54K, IFIT-2, G10P2, IFI54, P54, GARG-39, ISG-54K, IFI-54, CIG-42, Cig42*
*IFIT3*	3437	5411	ENSG00000119917	604650	*IFI-60K, CIG-49, IFIT-3, IFIT-4, ISG-60, IFIT4, CIG49, IFI60, RIG-G, P60, GARG-49, Cig41, IRG2*
*IFIT5*	24138	13328	ENSG00000152778	616135	*IFIT-5, ISG58, RI58, P58*

**Figure 2:**
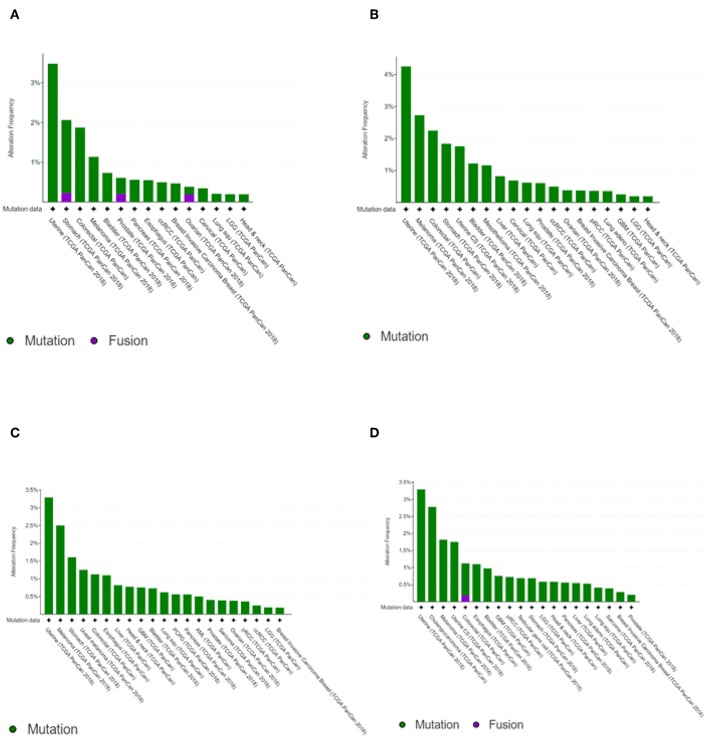
Cancer type summary of mutation frequencies in IFIT genes across the cancer studies. Pictures shows the histograms of alteration of mutation frequencies in *IFIT1* **(A)**, *IFIT2* **(B)**, *IFIT3* **(C)**, and *IFIT5* **(D)** across the cancer studies. Data obtained from TCGA PanCancer Atlas studies. Picture courtesy of cBioPortal.

The transcription kinetics of individual IFIT genes vary depending on the stimulus, exposure time, and cell or tissue type. For instance, human *IFIT1* expression is high at 6 h after IFN treatment and decreases rapidly within 12 to 24 h of treatment (Kusari and Sen, 1986). *IFIT1* mRNA levels are high in HT1080 fibrosarcoma cells 24 h after treatment with *IFN-*β, whereas *IFIT2* mRNA levels are substantially decreased (Terenzi et al., 2006). In HEK293 cells, the mRNA levels of both *IFIT1* and *IFIT2* remain high even after 24 h of treatment with IFN (Terenzi et al., 2006). However, anti-inflammatory therapies with glucocorticoids such as dexamethasone negatively regulate the transcription of IFIT genes due to the competitive binding of the glucocorticoid receptor with glucocorticoid receptor-interacting protein-1 (*GRIP1*), thereby inhibiting IRF-3 activation (Smith and Herschman, 1996; Reily et al., 2006). IFIT genes have been shown to undergo mutations. However, the mutation frequencies were relatively low compared to other cancer driver genes (Figure 2) (Cerami et al., 2012; Gao et al., 2013). In head and neck cancer, the mutation frequencies are lower than 1%. Since mutations in IFIT genes can alter the structure of encoded proteins and significantly affect protein stability and their interactions with other cellular proteins (Johnson et al., 2018), prior knowledge of mutations of IFIT genes may be crucial for investigating the cause of cancers.

### IFITs With Versatile TPR Motifs in Their Protein Structure

**Figure 3:**
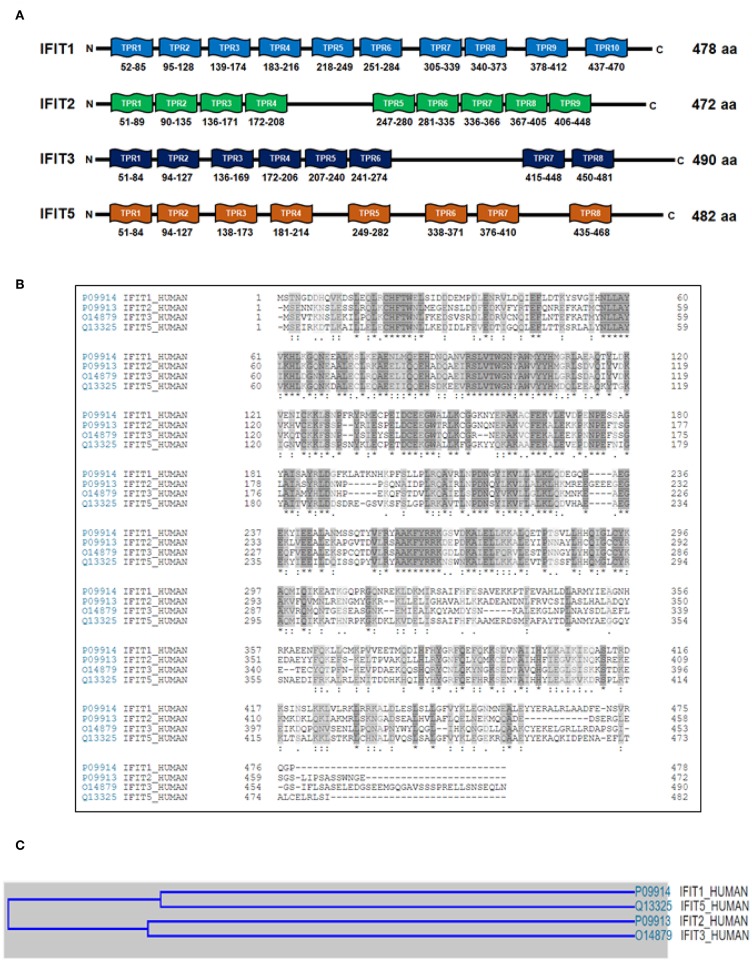
The organization of tetratricopeptide repeat (TPR) motifs and phylogenetic analyses of human IFIT family proteins. **(A)** The schematic illustration shows the predicted number of TPR motifs in *IFIT1* (P09914), *IFIT2* (P09913), *IFIT3* (O14879) and *IFIT5* (Q13325) according to the UniProtKB database. The numbers are UniProtKB accession numbers. **(B)** Sequence alignments of human IFIT proteins with Clustal Omega. The multiple-sequence alignment shows that *IFIT1, IFIT2, IFIT3*, and *IFIT5* share only 25% sequence similarity. **(C)** The cartoon depicts the evolutionary relationship among human IFIT proteins. The multiple-sequence alignment using Clustal Omega reveals that *IFIT1* and *IFIT5* have 54% sequence similarity, whereas *IFIT2* and *IFIT3* have 52%.

The molecular weights of the human IFIT proteins range from 54 to 60 kDa. Hence, *IFIT1, IFIT2, IFIT3*, and *IFIT5* proteins are denoted as p56, p54, p60, and p58, respectively; the number represents the molecular weight of the proteins (Table 2). The unique characteristic feature of IFIT proteins is that all of them contain many TPR motifs distributed across the whole sequence. IFIT proteins have different numbers of TPR motifs in their structure. The TPR is a structural unit of IFIT proteins comprised of 3 to 16 degenerate tandem repeats of 34 amino acids that form helix-turn-helix arrangements enabling them to be involved in protein-protein interactions and are frequently found in scaffold proteins (Allan and Ratajczak, 2011). The antiparallel helices of multiple TPR motifs impart a unique folding nature to the IFIT proteins that allow for the binding of distinct cellular proteins (Blatch and Lassle, 1999; D'Andrea and Regan, 2003). The proposed consensus amino acid sequence conventionally found in TPR motifs is W_4_G_8_Y_11_G_15_Y_17_A_20_Y_24_A_27_P_32_ (D'Andrea and Regan, 2003). However, TPRs are degenerate in amino acid sequence, and the tandem repeats of each amino acid residue are variable and can be replaced by other classes of amino acids. Therefore, the prediction of TPR motifs in a protein is difficult. According to the UniProtKB database, the predicted number of TPR motifs in human *IFIT1* (P09914), *IFIT2* (P09913), *IFIT3* (O14879), and *IFIT5* (Q13325) proteins is 10, 9, 8, and 8, respectively (Figure 3A). Although all human IFIT proteins share TPR motifs in their structure, the amino acid sequence similarity among *IFIT1, IFIT2, IFIT3*, and *IFIT5* is merely 25% (Figure 3B). Phylogenetic analysis of human IFIT proteins reveals evolutionary relationships between *IFIT1* and *IFIT5* and between *IFIT2* and *IFIT3* (Liu et al., 2013) (Figure 3C), whereas the sequence identity is 54% between *IFIT1* and *IFIT5* and 52% between *IFIT2* and *IFIT3*. This implies that each IFIT protein may have unique biological functions, which is indeed witnessed in the cellular and molecular functions of different IFIT-family proteins. IFIT proteins such as *IFIT1, IFIT2*, and *IFIT3* can form homo-oligomers in solutions (Pichlmair et al., 2011), except for *IFIT5*, which is solely monomeric. The crystal structure of human *IFIT2* has shown that it makes homo- or heterodimers with other IFIT family members (Stawowczyk et al., 2011; Sen and Fensterl, 2012). Although the complete crystal structure of *IFIT3* is not available, *IFIT3* may be similar to *IFIT2* in forming a homodimer because the N-terminal domain of *IFIT3* has a 70% sequence similarity with *IFIT2* (Yang et al., 2012). The first protein complex of *IFIT1, IFIT2*, and *IFIT3* was found in the IFN-treated HeLa cell lysates (Stawowczyk et al., 2011). Subsequently, protein pulldown assays followed by mass spectrometry analysis have shown that IFIT proteins could associate with other members of the IFIT family except *IFIT5* (Pichlmair et al., 2011; Habjan et al., 2013). In addition, we observed the protein complex of *IFIT1, IFIT2*, and *IFIT3* in OSCC cells by co-immunoprecipitation assay (Pidugu et al., 2019b). These findings suggest that *IFIT1, IFIT2*, and *IFIT3* can form protein complexes in cells. Recent research revealed that *IFIT1, IFIT2*, and *IFIT3* proteins interact with each other via a conserved YxxxL motif in the C-terminus of each protein. In the functional complexity of these three proteins, *IFIT3* acts as a central scaffold and regulates the functions of *IFIT1* and *IFIT2* (Kumar et al., 2014; Fleith et al., 2018; Mears and Sweeney, 2018). Recent protein crystallographic studies of *IFIT1* and *IFIT5* have also revealed that IFITs can interact with viral RNA containing 5' triphosphate group (PPP-RNA) (Abbas et al., 2013; Feng et al., 2013; Katibah et al., 2013). Furthermore, the crystal structure reveals that *IFIT2* specifically binds with AU-rich RNAs (Sen and Fensterl, 2012; Yang et al., 2012).

**Table 2:** Summary of Human IFIT gene encoded proteins.

**Gene name**	**Protein name**	**UniprotKB ID**	**No. of amino acids**	**No. of predicted TPR motifs**
*IFIT1*	P56	P09914	478	10
*IFIT2*	P54	P09913	472	9
*IFIT3*	P60	O14879	490	8
*IFIT5*	P58	Q13325	482	8

### Interdependence Between IFIT1 and IFIT3

Protein sequence analysis revealed that *IFIT1* and *IFIT3* share only 37% sequence similarity. Intriguingly, the expression of *IFIT1* was strongly correlated with *IFIT3* in both OSCC cell lines and tissues derived from OSCC patients (Pidugu et al., 2019b). Ectopic expression of *IFIT3* also induced endogenous *IFIT1* expression in a dose-dependent manner (Johnson et al., 2018). These data indicate that *IFIT1* and *IFIT3* are interdependent. This was further supported by the studies where deletion or mutation in the C-terminal YxxxL motif of *IFIT3* resulted in decreased *IFIT1* expression (Fleith et al., 2018). This could be due to the enhanced *IFIT1* protein stability or decreased degradation by *IFIT3* in the complex. Alternatively, *IFIT3* may promote *IFIT1* activity by enhancing the *IFIT1* concentration in the cell by averting its turnover by locking it in a stable complex (*IFIT1: IFIT3*) (Fleith et al., 2018; Johnson et al., 2018). Formation of the complex with *IFIT3* also seems to increase the *IFIT1* specificity, since *IFIT3* binds with *IFIT1* and facilitates the binding of *IFIT1* with Cap0 RNAs, and hence functional studies demonstrated that *IFIT3* is required for the stabilization of *IFIT1* expression and its antiviral functions in the cell (Johnson et al., 2018). *IFIT1* is phosphorylated at 24 h after Sendai virus infection in HEK293 cells, yet the phosphorylation site and its subsequent effect of phosphorylation remain elusive (Li et al., 2009). Since the tyrosine in the *IFIT3* C-terminus YxxxL motif is crucial for the interaction between *IFIT1* and *IFIT3* (Fleith et al., 2018), it would be noteworthy to determine whether this tyrosine residue is prone to phosphorylation and how this impacts *IFIT1: IFIT3* complex formation. Moreover, it was found that a small proportion of *IFIT1* was ISGylated by human ISG15 protein in IFN-β-treated HeLa cells; however, the consequences of this modification remain unclear, and further studies are warranted to understand the mechanistic insights into *IFIT1: IFIT3* complex formation (Zhao et al., 2005).

### IFITs in Malignant Progression

Although IFITs are involved in the host immune response and antiviral defense, emerging research studies have shown their involvement in malignant progression (Lai et al., 2008, 2013; Niess et al., 2015; Zhang et al., 2016; Ohsugi et al., 2017; Yang et al., 2017; Zhao et al., 2017; Chen et al., 2018; Lo et al., 2018, 2019; Nushtaeva et al., 2018; Shen et al., 2018; Huang et al., 2019). The epithelial-mesenchymal transition (EMT) is a biological mechanism in which epithelial cells convert into a mesenchymal phenotype to acquire increased cell invasion and migration capacity as well as resistance to apoptosis (Kalluri and Weinberg, 2009), which lead to metastasis (Yilmaz and Christofori, 2009). Accumulated evidence has shown that EMT is linked to an increased risk of cancer invasion and decreased the survival rate of OSCC patients (Yilmaz and Christofori, 2009; Natarajan et al., 2014). In our previous studies, we found an inverse association of *IFIT2* with the migration activity of OSCC cells (Lai et al., 2008) and showed reduced *IFIT2* expression level was correlated with an increased rate of metastasis in OSCC patients (Lai et al., 2013). *IFIT2* exhibited distinct cytoskeletal staining patterns in squamous cells and surrounding normal cells in OSCC patient tissues and interacted with cytokeratins such as *CK8* and *CK18*. Knockdown of endogenous *IFIT2* expression using *IFIT2*-specific siRNA enhanced OSCC cell migration rates. Strikingly, high *IFIT2* expression in tumor tissues was correlated with better patient survival (Lai et al., 2008). The enhanced malignancy of *IFIT2*-depleted OSCC cells was attributed to increased expression and secretion of tumor necrosis factor-alpha (*TNF-*α), and hence blocking *TNF-*α abolished the angiogenic activity of *IFIT2-*silenced metastatic cells (Li et al., 2012). We also demonstrated that knockdown of *IFIT2* induced EMT in OSCC by activating the atypical *PKC* signaling pathway (Lai et al., 2008, 2013).

We recently found that ectopic overexpression of *IFIT1* and *IFIT3* enhanced *EGFR* and *AKT* activation and subsequently promoted OSCC invasion through EMT (Pidugu et al., 2017). *IFIT1* expression was strongly correlated with *IFIT3* expression in OSCC cell lines and patient tissues. Additionally, the expression of both *IFIT1* and *IFIT3* was associated with phospho-EGFR in OSCC specimens. Clinicopathological and survival analyses showed that elevated *IFIT1* and *IFIT3* expression correlated with poor survival in OSCC patients. Furthermore, we demonstrated that *IFIT1* and *IFIT3* enhanced the *EGFR* endocytic recycling process by interacting with Annexin-2 (*ANX2*) (Pidugu et al., 2019b). However, *IFIT3* has been shown to have antiproliferative activity by enhancing the expression of cell cycle negative regulators such as *p27* and *p21* in monocytic U937 cells (Xiao et al., 2006). *IFIT3* increased *p21* protein level by downregulating *c-Myc*, a repressor of *p21* in the cell (Xiao et al., 2006). IFIT proteins execute multiple complex cellular functions based on cell type and tissue types. Therefore, the functions of IFIT proteins could be altered depending on the cell system. Hence, the controversial functions of *IFIT3* warrant further investigation. *IFIT5* has been implicated exclusively in an innate immune response. However, a newly identified mechanism of *IFIT5* is regulation of the turnover of tumor suppressor microRNAs (miRNAs), including *miR-363* and *miR-128*, resulting in increased expression of transcription factors of EMT such as *slug* and *ZEB1*, thereby enhancing invasion in renal cell carcinoma (RCC) (Lo et al., 2019). Moreover, the oncogenic role of *IFIT5* has been identified in bladder cancer. *IFIT5* promoted cell invasion and migration by inducing EMT by downregulating *miR-99a* in bladder cancer (Huang et al., 2019). *IFIT5* expression was also inversely correlated with *miR-363* expression in prostate cancer. *IFIT5* is involved in the degradation of *miR-363* and can form a complex with *miR-101* and *miR-128* to promote prostate cancer progression by inducing EMT (Lo et al., 2018).

### IFITs in Apoptosis

Intriguingly, IFIT-family proteins display distinct functions in apoptosis. The negative association of *IFIT2* with tumor malignancy is likely due to its pro-apoptotic activity (Stawowczyk et al., 2011; Chen et al., 2017). Ectopic *IFIT2* overexpression induced the activation of caspase-3 and disturbed the plasma membrane asymmetry and permeability, which is a basic characteristic feature of apoptosis (Stawowczyk et al., 2011; Lai et al., 2013; Feng et al., 2014). In addition, *IFIT2* was associated with the mediator of IRF3 activation (MITA) and regulated apoptotic cell death via the mitochondrial pathway (Stawowczyk et al., 2011). Inhibition of proteasome-mediated degradation of *IFIT2* led to the aggregation of *IFIT2* in the perinuclear region and promoted apoptosis (Chen et al., 2017). Derepression of *IFIT2* made the cells prone to apoptotic death induced by external stimuli such as chemotherapeutic drugs and serum starvation (Feng et al., 2014; Wang et al., 2016). *IFIT2*-mediated apoptosis was not dependent on the DNA damage response (Chen et al., 2017; Ohsugi et al., 2017). Rather, *IFIT2* induced apoptosis by regulating the balance between pro- and anti-apoptotic Bcl-2 family proteins, which altered the permeability of the mitochondrial membrane (Tait and Green, 2010; Stawowczyk et al., 2011). Several research studies have confirmed the ability of *IFIT2* to promote the apoptotic death of cancer cells, including OSCC (Lai et al., 2013; Feng et al., 2014), colorectal cancer (Jia et al., 2017; Ohsugi et al., 2017), leukemia (Zhang et al., 2017), osteosarcoma (Wang et al., 2016), and hepatocellular carcinoma (Tang et al., 2017).

*IFIT3* acts as a bridging molecule between the mitochondrial antiviral signaling (MAVS) complex and the TNFR-associated factor family member-associated *NF-*κ*B* activator binding kinase 1 (*TBK1*) to regulate the activation of *IRF3* and *NF-*κ*B* (Liu et al., 2011). Overexpression of *IFIT3* had a protective role in human lung epithelial cells, whereas depletion of *IFIT3* expression induced apoptotic cell death (Hsu et al., 2013). Studies have shown that co-expression of *IFIT3* inhibited *IFIT2*-dependent apoptotic cell death (Stawowczyk et al., 2011). Since *IFIT2* and *IFIT3* form a protein complex in the cell, the apoptotic effects of *IFIT2* can be negatively regulated by *IFIT3* (Reich, 2013). However, further studies are needed to understand whether the pro-survival signal of *IFIT3* alone can protect the cell or whether the *IFIT2: IFIT3* association is required to modulate *IFIT2*-mediated apoptosis.

*IFIT1* negatively regulated *NF-*κ*B* and *IRF3* activation by interrupting the *MITA-MAVS-TBK1* interaction in the cell (Li et al., 2009). Our recent study has shown that *IFIT1* interacted with *IFIT3* and that *IFIT1* and *IFIT3* expression promoted OSCC cell proliferation and metastasis (Pidugu et al., 2019b). However, the anti-apoptotic roles of *IFIT1/IFIT3* have to be investigated in OSCC. Ectopic expression of *IFIT5* induced *IRF-3*- and *NF-*κ*B*-mediated gene expression. Furthermore, *IFIT5* colocalized with *MAVS* (Zhang et al., 2013; Zheng et al., 2015). Recent research has shown that *IFIT5* promoted the progression of various cancers, including renal cancer, prostate cancer, and bladder cancer (Lo et al., 2018, 2019; Huang et al., 2019). Hence, it would be interesting to investigate whether IFIT5 may also have anti-apoptotic functions.

### IFITs in Drug Resistance

The failure of chemotherapy or targeted therapy has often been associated with intrinsic or acquired drug resistance in cancer cells (Mansoori et al., 2017). Anticancer drug resistance can be caused by various intrinsic cellular mechanisms, including alterations of intracellular drug distribution, changes in drug metabolism, decreased apoptosis, enhanced drug efflux by ATP-binding cassette transporters, enhanced DNA damage repair, cell cycle dysregulation, and reduced drug-target interactions (Larsen et al., 2000). Increased expression of *IFIT1* and *IFIT3* genes was observed in DNA damage-resistant sublines compared to parental cell lines in various cancers, including OSCC cells (Weichselbaum et al., 2008). *IFIT1* and *IFIT3* were also upregulated in estrogen-negative breast cancer cells in post-chemotherapy residual tumors (Legrier et al., 2016). Overexpression of *IFIT1* or *IFIT3* increased OSCC resistance to various chemotherapeutic drugs, including cisplatin, carboplatin, oxaliplatin, 5FU, and ganetespib. Interestingly, *IFIT1* and *IFIT3* expression made OSCC cells susceptible to gefitinib (EGFR-TKI) (Yen et al., 2014; Pidugu et al., 2018, 2019a). *IFIT1* and *IFIT3* promoted *EGFR* activation in OSCC cells and enhanced the tumor-preventive activity of gefitinib. In addition, the combination treatment of *IFN-*α and gefitinib showed synergistic anti-tumor activity in OSCC cells (Bruzzese et al., 2006; Pidugu et al., 2019b). These studies demonstrated that *IFIT1* and *IFIT3* modulate the drug response via *EGFR* signaling. The efficacy of the therapies with various drugs depends on the cellular mechanisms active in the cancer cells. Thus, advanced metastatic OSCC must be treated with multifaceted therapies to improve the clinical outcome in patients. Therefore, targeting specific IFITs can be a good clinical approach for OSCC treatment. We have also shown that *IFIT2* knockdown enhances atypical *PKC* signaling in OSCC cells (Lai et al., 2013). Activation of *PKC* signaling is involved in the development of multidrug resistance (MDR) phenotype by phosphorylation of p-glycoprotein in cancer cells (Rumsby et al., 1998). On the other hand, inhibition of atypical *PKC* has improved clinical outcomes in advanced basal cell carcinoma (BCCs) (Mirza et al., 2017). Therefore, *IFIT2* depletion may promote drug resistance. Hence, additional studies are warranted to determine the *IFIT2*-mediated drug resistance in OSCC.

### IFITs in HVP Infection

The HPV infection is one of the aetiological factors of OSCC pathogenesis (Gillison et al., 2008; Husain and Neyaz, 2017). Studies have shown that OSCC patients with HPV infection had a better prognosis compared to patients with no infection (Schwartz et al., 2001). However, the biological mechanism that leads to better clinical outcomes in OSCC patients is not clear. *IFIT1* has been shown to act against HPV by direct binding with key E1 replication protein, which is crucial for the synthesis of viral progeny DNA. The HPV E1 protein binds at the viral origin of replication DNA elements and makes a hexameric helicase complex with the help of HPV E2 protein. This protein complex interacts with several host cell proteins such as DNA polymerase alpha and recruits replication protein A (RPA) to initiate viral DNA replication (Wilson et al., 2002). The binding of the N-terminus of human *IFIT1* with HPV E1 inhibits its helicase function and stops viral DNA replication by uncoupling it from the viral DNA. It has been demonstrated that *IFIT1* binding activity with HPV E1 is not virus strain-specific, as it binds with E1 of HPV11, HPV18, and HPV31. HPV replication is inhibited to a great extent by the treatment with interferons, and this effect is weakened by the shRNA-mediated knockdown of *IFIT1*. Thus, human *IFIT1* is considered the prime antiviral protein against HPV (Terenzi et al., 2008).

Human *IFIT5* plays a crucial role in the host innate immune response during viral infection (Zhang et al., 2013; Zheng et al., 2015). Interestingly, we found that the expression of human *IFIT5* protein is highly correlated with HPV E6 protein in tissues of OSCC patients (Yen et al., 2014). However, the underlying molecular mechanism involved in the association between *IFIT5* and HPV E6 proteins is not yet understood. HPV E6 is one of the major oncoproteins that contribute to the malignant progression of OSCC. One of the major functions of HPV E6 is to enhance the proteasome-mediated degradation of tumor suppressor protein p53 via the interaction with E6-associated protein (*E6AP*). *p53* regulates cell growth and apoptosis immediately after DNA damage. HPV E6 also impedes pro-apoptotic proteins, such as procaspase 8 and *Bak*, to block apoptosis (Mantovani and Banks, 2001; Gupta and Gupta, 2015). Over the past decade, an increasing number of HPV E6-associated proteins have contributed to cellular malignant transformation, with human telomerase as one of the best examples (Narisawa-Saito and Kiyono, 2007). Recently, human *IFIT5* has been implicated in cancer progression (Lo et al., 2018, 2019; Huang et al., 2019). Therefore, the correlation between human *IFIT5* and HVP E6 might contribute to OSCC malignancy, and further investigation is warranted to explore the reason for their association.

### IFITs as Putative Co-chaperones

Heat shock protein 90 (*Hsp90*) is a ubiquitously expressed ATPase-directed molecular chaperone in the cell, which facilitates posttranslational protein homeostasis by regulating a variety of molecular processes such as stabilization, maturation, degradation of client proteins that are involved in various signal transduction pathways (Caplan, 1999; Whitesell and Lindquist, 2005; Pearl and Prodromou, 2006). Hsp90 regulates the folding and activation of more than 200 client proteins (https://www.picard.ch/downloads/Hsp90interactors.pdf), including *EGFR* (Ahsan et al., 2012), *AKT* (Basso et al., 2002), *SAPK* (Tatebe and Shiozaki, 2003), *p38* (Ota et al., 2010), *PKC* (Gould et al., 2009), *FAK* (Xiong et al., 2014), and *DNA-PK* (Solier et al., 2012). *Hsp90* controls the maturation and intracellular trafficking of ErbB2-family proteins, including *EGFR* (Xu et al., 2001, 2002; Yang et al., 2016). The biological function of *Hsp90* relies on an inherent ATPase activity that is modulated by many of its co-chaperones. Emerging evidence has indicated that many TPR proteins, such as Hsp90-organizing protein (*HOP*), carboxy-terminus of Hsc70–interacting protein (*CHIP*), *Cdc37, PP5, Unc45, Fkbp51, Fkbp51, Fkbp52, Sti1*, and *Cyp40*, act as co-chaperones and play crucial roles in stabilizing the interaction between chaperones, such as *Hsp90* or *Hsp70*, and their client proteins (Bose et al., 1996; Frydman and Hohfeld, 1997; Chen et al., 1998; Blatch and Lassle, 1999; Caplan, 1999; Scheufler et al., 2000; Li et al., 2012). *HOP* interacts with *Hsp70* and *Hsp90* with its TPR1 and TPR2A domains, respectively. Both TPR1 and TPR2A domains of *HOP* bind with EEVD motifs of *Hsp70* and *Hsp90* through electrostatic interactions between C-terminal aspartates and hydrophobic interactions of amino acid residues upstream of the EEVD motifs (Chen and Smith, 1998; Scheufler et al., 2000; Brinker et al., 2002; Travers and Fares, 2007). *CHIP*, a quality-control E3 ligase containing a co-chaperone protein, regulates the degradation of ubiquitinylated client proteins, which is crucial for maintaining protein turnover and cellular homeostasis. *CHIP* has three N-terminal tandem repeats of TPR motifs by which it interacts with and regulates *Hsp70* and *Hsp90* (Ballinger et al., 1999; Paul and Ghosh, 2014). Intriguingly, the balance between client protein folding and degradation is regulated by the binding of *HOP* or *CHIP* with *Hsp90* and *Hsp70*. Emerging evidence has shown that the proteins with TPR motifs bind a C-terminal highly conserved motif, the EEVD-COOH tail, of *Hsp70/Hsp90* to control protein folding and maturation (Demand et al., 1998; Scheufler et al., 2000; Brinker et al., 2002). Furthermore, the binding of these co-chaperones is determined by the C-terminal phosphorylation status of *Hsp70* and *Hsp90* (Muller et al., 2013). Since IFIT proteins contain multiple TPR motifs in their structures, we speculated that they may exert co-chaperone functions. *Hsp90* has two isoforms, *Hsp90*α and *Hsp90*β, but the functional difference between these isoforms is not well-understood (Subbarao Sreedhar et al., 2004). Interestingly, co-immunoprecipitation followed by LC-MS/MS analysis showed 16 peptides with 21% sequence coverage and 17 peptides with 16% sequence coverage of Hsp90α; 59 peptides with 50% sequence coverage and 57 peptides with 44% sequence coverage of *Hsp90*β were identified with immunoprecipitates of *IFIT1* and *IFIT3*, respectively. Protein-protein interaction network analysis using the STRING 10.5 database showed that heat shock protein 90 s (*Hsp90*α*/*β) directly bound *IFIT1* and *IFIT3* (Pidugu et al., 2019a,b). Increased C-terminal phosphorylation of Hsp90α was observed in *IFIT1*- or *IFIT3*-overexpressing OSCC cells. Overexpression of *IFIT1* or *IFIT3* in OSCC cells enhanced the phosphorylation of *Hsp90* client proteins, including *EGFR, AKT, p38*, and *SAPK/JNK*. Alternatively, inhibition of *Hsp90* activity by ganetespib led to decreased C-terminal phosphorylation of *Hsp90*α and expression and activation of its downstream client proteins (Pidugu et al., 2019a). These *Hsp90*-downstream signaling regulators have been widely shown to play crucial roles in cell survival, migration, invasion, and metastasis (Tsutsumi and Neckers, 2007; Pashtan et al., 2008; Tsutsumi et al., 2009). Since *IFIT1* and *IFIT3* contain TRP motifs, we may hypothesize that they function as co-chaperones. Further investigation is warranted to elucidate the TPR motifs of *IFIT1* and *IFIT3* that mediate protein-protein interactions in OSCC. A recent study has shown that *IFIT2* is co-precipitated with *p67phox* and mitochondria-associated heat shock protein *Hsc70* (Stawowczyk et al., 2018). As *IFIT2* contains many TPR domains in its structure, it binds with *p67phox* and *Hsc70* through TPR motif-mediated interactions; however, the exact TPR motifs involved in the interaction have not yet been mapped. Since increased *Hsp90* activity directly influences client kinase activation and stabilization, Hsp90 is regarded as a promising therapeutic target for cancer treatment (Caplan et al., 2007; Trepel et al., 2010). Further studies are needed to delineate the co-chaperone functions of IFITs in the future.

### The Clinical Relevance of IFITs in OSCC and Other Cancers

The ISGs and their signaling pathways play vital roles in the malignant transformation of cells in the tumor microenvironment. Although IFNs have been used as exogenous pharmaceuticals for the treatment of cancers, paradoxical findings revealed that constitutive expression of aberrantly regulated ISGs promotes neoplastic disease development and progression (Cheon et al., 2014). Abnormal expression of ISGs promotes tumor invasion and progression in many cancers, including skin cancer, breast cancer and head and neck cancers (Perou et al., 1999; Suomela et al., 2004; Andersen and Hassel, 2006; Hatano et al., 2008). Increased expression of ISGs has been reported in metastatic cancer cells compared to non-metastatic cells (Cai et al., 2009; Khodarev et al., 2009). ISGs can modulate the tumor cell response to therapeutic drugs by altering the tumor microenvironment. Defects in interferon signaling cause resistance to immunotherapies such as anti-CTLA-4 and PD1 blockade (Shin et al., 2015; Gao et al., 2016; Zaretsky et al., 2016). Constant exposure to low levels of IFNs induces the transcription of a subset of ISGs called interferon-related DNA damage-resistant signature (IRDS) genes that contribute to tumor growth, metastasis, resistance to radiation therapy and chemotherapeutic drugs (Rickardson et al., 2005; Duarte et al., 2012; Cheon et al., 2013). High expression of IRDS genes in cancer tissues promotes tumor growth, invasion, and metastasis (Wallace et al., 2011). *IFIT1* and *IFIT3* genes are classified as IRDS subset genes and are considered as predictive biomarkers for chemotherapy and radiation therapy in many primary human cancers, such as breast cancer, head and neck cancer, prostate cancer, lung cancer, and glioma (Weichselbaum et al., 2008). Therefore, cancer patients with IRDS expression are suggested to undergo therapies combined with adjuvant treatments. Accordingly, high *IFIT1* and *IFIT3* expression have been correlated with a better therapeutic response to IFNs along with chemotherapeutics and immunostimulating agents in patients with breast cancer, glioblastoma, and hepatocellular carcinoma (Zhang et al., 2016; Yang et al., 2017; Nushtaeva et al., 2018). However, the enhanced expression of *IFIT3* in pancreatic cancer has been shown to cause pseudo-inflammation and result in cancer progression (Niess et al., 2015; Zhao et al., 2017). Our recent investigations also demonstrated that high *IFIT1* or *IFIT3* expression correlated with T-stage, lymph node metastasis, lymphovascular, perineural invasion, and poor overall survival in OSCC patients. Ectopic expression of *IFIT1* or *IFIT3* in OSCC cells promoted tumor growth and metastasis by activating *EGFR* signaling (Pidugu et al., 2019b). Strikingly, we also found that enhanced *IFIT1* or *IFIT3* expression in OSCC cells promoted sensitivity to gefitinib (EGFR-TKI). Moreover, the combination treatment of gefitinib and *IFN-*α resulted in a synergistic tumor-inhibitory effect in OSCC (Bruzzese et al., 2006; Pidugu et al., 2018). This could be due to the enhanced *EGFR* tyrosine kinase activity in OSCC cells caused by *IFIT1* and *IFIT3* expression. These data suggest that *IFIT1* and *IFIT3* expression can be used as prognostic biomarkers to predict the EGFR-TKI and *IFN-*α therapeutic response in OSCC patients and probably in patients with other cancer types.

Interestingly, we also observed that high *IFIT1* or *IFIT3* expression in OSCC cells increased resistance to various therapeutics, including cisplatin, oxaliplatin, carboplatin, 5-FU, and ganetespib. *IFIT1* or *IFIT3* expression enhanced C-terminal phosphorylation of *Hsp90* and its client proteins, including *PKC, EGFR, AKT*, and *p38* (Pidugu et al., 2017, 2019a). Protein-protein interaction analysis revealed that *IFIT1* and *IFIT3* colocalize with both Hsp90α and *Hsp90*β isoforms in OSCC cells. Collectively, these data suggest that *IFIT1* and *IFIT3* regulate the drug response and might function as co-chaperones in OSCC. Thus, *IFIT1* and *IFIT3* proteins can be considered potential therapeutic targets for OSCC. In contrast, *IFIT2* has been shown to have tumor suppressor function in many cancers. Decreased expression of *IFIT2* is associated with increased cell proliferation and metastasis and predicts poor clinical outcome in gastric cancer patients (Chen et al., 2018). Downregulation of *IFIT2* expression was observed in colorectal cancer (CRC) tissues compared to normal tissues. Overexpression of *IFIT2* in CRC cells suppressed cell growth and increased apoptosis (Ohsugi et al., 2017). Our findings agree with previous findings that *IFIT2* depletion significantly increased OSCC metastasis both *in vitro* and *in vivo* (Lai et al., 2013). Besides, the high expression of *IFIT2* in OSCC tumor tissues negatively correlated with the nodal stage. The positive association of *IFIT2* expression with better overall patient survival suggests that *IFIT2* can act as a novel prognostic biomarker to predict OSCC progression (Lai et al., 2008). Although *IFIT2* belongs to the same family, it executes tumor suppressor functions, whereas *IFIT1* and *IFIT3* have tumor-promoting properties in OSCC. However, how IFITs regulate contradictory functions in OSCC cells is not yet known. Further investigations are required to understand the possible molecular mechanisms that regulate opposing functions of IFITs in OSCC.

## Discussion

IFITs are quickly induced by multiple stimuli, such as IFN-dependent or IFN-independent signaling pathways. Over the past decade, the anti-viral functions of IFITs have been extensively studied. Many structural and functional studies propose that IFITs are also involved in the regulation of cell-intrinsic and cell-extrinsic immune responses via new pathways that must be corroborated. Although IFITs have a simple gene architecture and promoter structure, they execute multiple complex functions based on stimulus, cell type, and tissue type. The variable amino acid sequence in TPR motifs could be the reason for the broad range of non-redundant functions mediated by IFITs in the cell. Even though IFITs are renowned antiviral proteins, recent studies hint at their complex biological and molecular roles in cancer.

**Figure 4:**
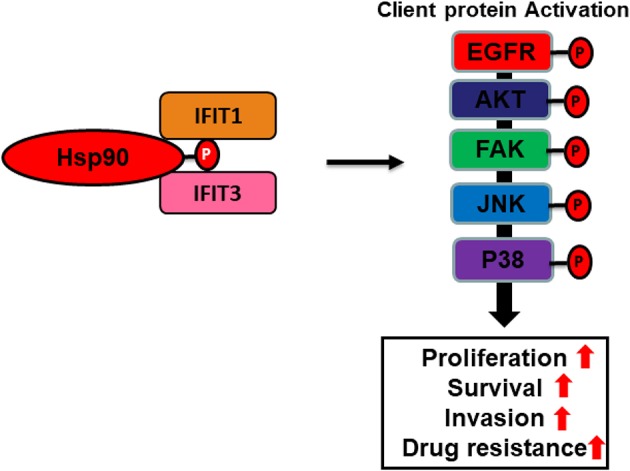
Cartoon illustration depicting putative co-chaperone functions of IFIT1 and IFIT3. A hypothetic scheme shows that *IFIT1/IFIT3* may regulate C-terminal phosphorylation of *Hsp90* and hence enhance proper folding and maturation of its downstream signaling regulators in OSCC.

In this review, we hypothesize that IFIT protein may serve as co-chaperones of Hsp90 (Figure 4). Many studies have demonstrated that TPR proteins interact with the C-terminus of *Hsp90* to facilitate the stabilization of the chaperone complex (Scheufler et al., 2000; Brinker et al., 2002; Muller et al., 2013). *Hsp90* is a ubiquitously expressed molecular chaperone in the cells that regulates a multitude of cellular processes, such as client kinase folding, maturation, and degradation (Whitesell and Lindquist, 2005). The direct association of *IFIT1* or *IFIT3* with heat shock proteins (*Hsp90*α*/*β) suggests a unique mechanism of action of *IFIT1* and *IFIT3* that may be involved in activating *Hsp90* and several of its downstream signaling regulators, which are crucial for OSCC tumor progression and drug resistance. Thus, *IFIT1* and *IFIT3* may likely function as co-chaperones and serve as potentially important therapeutic targets for OSCC.

**Figure 5:**
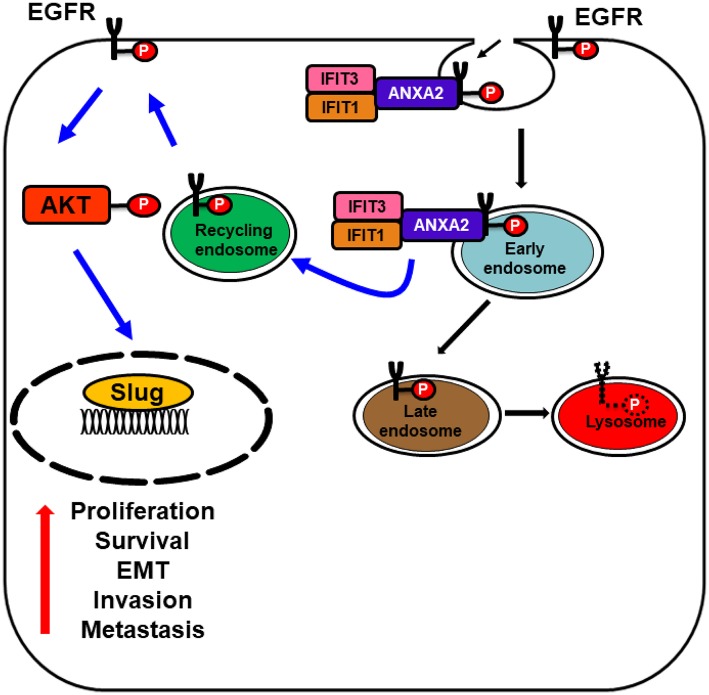
Schematic model represents IFIT1 and IFIT3 interaction with ANXA2 promote EGFR recycling. Diagram illustrating the association of *IFIT1* and *IFIT3* with *ANXA2* and enhance p-EGFRY^1068^ endocytic recycling, which subsequently leads to *EGFR, AKT* activation, and enhance the expression of downstream epithelial-mesenchymal transition (EMT) transcription factor *slug*. Activated *EGFR* and its downstream signaling regulators promote OSCC invasion and metastasis.

Alternatively, our recent studies have shown that ectopic overexpression of *IFIT1* and *IFIT3* enhanced the *EGFR* endocytic recycling process by interacting with Annexin-2 (*ANX2*) recycling and thus activated several downstream pathways involving in cell proliferation, survival, and drug resistance. Activated EGFR subsequently promoted OSCC invasion through EMT (Figure 5). This hypothesis was supported by the clinical observations showing a positive association of phospho-EGFR levels with the expression of both *IFIT1* and *IFIT3* in OSCC specimens. In addition, elevated *IFIT1* and *IFIT3* expression indeed correlated with poor survival in OSCC patients. In contrast, decreasing *IFIT2* expression activated PKC pathway and promoted the progression of tumor malignancy. However, how PKC is activated by depleting IFIT2 protein is still unknown. It cannot be neglected that IFIT proteins may execute controversial activity in different cell types. However, we may infer that IFITs play certain roles on modulation of survival signaling pathways.

Additionally, recent studies have shown that *IFIT1* undergoes ISGylation (Zhao et al., 2005) and phosphorylation (Li et al., 2009), indicating that IFIT protein stability and function can be regulated by posttranslational modification. The association between *IFIT5* and HPV E6 indicates that *IFIT5* may be involved in the malignant transformation of oral squamous cells during disease progression. Additional studies are warranted to ascertain the role of *IFIT5* in HPV-positive OSCC.

In overall, the involvement of IFITs in the progression of cancer is an emerging question that warrants our concern. Apparently, these non-enzymatic proteins may exert their biologic activities via protein-protein interaction. Interruption of protein-protein interaction is a new clue for anti-cancer drug development. We prospect that *in vivo* studies with individual IFIT gene knockout mice can contribute to unraveling the pathophysiological roles of the respective IFIT proteins, and they could also open a new avenue for research into multiple disease systems and drug development in the future.

## Author Contributions

VP contributed to conception and manuscript writing. HP and M-MW contributed to the literature review. T-CL and C-JL defined the scope of the review and edited the manuscript. VP, HP, and T-CL revised the manuscript. All authors have read and approved the final manuscript.

### Conflict of Interest

The authors declare that the research was conducted in the absence of any commercial or financial relationships that could be construed as a potential conflict of interest.
